# Experimental and Simulation Studies of HPAM Microcomposite Structure and Molecular Mechanisms of Action

**DOI:** 10.3390/polym17223005

**Published:** 2025-11-12

**Authors:** Xianda Sun, Qiansong Guo, Yuchen Wang, Chengwu Xu, Wenjun Ma, Tao Liu, Yangdong Cao, Mingming Song

**Affiliations:** 1 State Key Laboratory of Continental Shale Oil, Northeast Petroleum University, College Station, Daqing 163318, China; sunxianda@nepu.edu.cn (X.S.); 18806351958@163.com (Y.W.); xuchw@nepu.edu.cn (C.X.); 2 Key Laboratory for Enhanced Oil & Gas Recovery of the Ministry of Education, Northeast Petroleum University, College Station, Daqing 163318, China; 18446296533@163.com (W.M.); 19214590395@163.com (T.L.); cyangdong0221@163.com (Y.C.); qaz154818967@163.com (M.S.)

**Keywords:** surfactant–polymer interaction, molecular dynamics simulation, electrostatic interaction, surface morphology, enhanced oil recovery (EOR)

## Abstract

Continental high water-cut reservoirs commonly exhibit strong heterogeneity, high viscosity, and insufficient reservoir drive, which has motivated the deployment of polymer-based composite chemical flooding, such as surfactant–polymer (SP) and alkali–surfactant–polymer (ASP) processes. However, conventional experimental techniques have limited ability to resolve intermolecular forces, and the coupled mechanism linking “formulation composition” to “microstructural evolution” remains insufficiently defined, constraining improvements in field performance. Here, scanning electron microscopy (SEM), backscattered electron (BSE) imaging, and molecular dynamics (MD) simulations are integrated to systematically investigate microstructural features of polymer composite systems and the governing mechanisms, including hydrogen bonding and electrostatic interactions. The results show that increasing the concentration of partially hydrolyzed polyacrylamide (HPAM) promotes hydrogen bond formation and the development of network structures; a moderate amount of surfactant strengthens interactions with polymer chains, whereas overdosing loosens the structure via electrostatic repulsion; the introduction of alkali reduces polymer connectivity, shifting the system toward an ion-dominated dispersed morphology. These insights provide a mechanistic basis for elucidating the behavior of polymer composite formulations, support enhanced chemical flooding performance, and ultimately advance the economic and efficient development of oil and gas resources.

## 1. Introduction

Most Chinese oilfields are characterized by continental depositional settings, pronounced reservoir heterogeneity, and weak natural drive. Combined with crude oils that are richer in wax and aromatics and of higher density, these factors constrain conventional waterflooding, leaving roughly two-thirds of the in-place reserves unrecovered [1,2,3,4,5]. In recent years, mature oilfields in eastern China have experienced declining production and persistently high water cut [6,7,8,9], revealing a mismatch between reserves and production capacity, increased difficulty in sustaining output, and pressure on project economics [10,11,12,13]. Against this backdrop, polymer systems are employed to varying extents in both conventional sandstone reservoirs and hydraulically fractured unconventional shale reservoirs; among them, partially hydrolyzed polyacrylamide (HPAM) is widely used owing to its relatively low cost and strong thickening capability. Chemical enhanced oil recovery based on HPAM and its composite formulations can modulate mobility and improve displacement efficiency [14,15,16,17,18]. However, achieving repeatable enhancement under conditions of high water content and strong heterogeneity still requires an experimentally verifiable microscopic mechanism framework specific to the formulation, in order to support formulation design and on-site parameter selection [19,20,21,22,23,24].

Most existing studies focus separately on displacement-scale behavior or molecular interactions, without linking formulation composition, microstructure, and molecular mechanisms into a coherent framework. Li et al. [25] enhanced polymer viscoelasticity and interfacial activity via molecular self-assembly and evaluated polymer-alkali and surfactant (ASP) performance at the macro scale, yet the microstructure of composite networks was not quantified. Li et al. [26] used visual micromodels to elucidate ASP synergy and reported improved recovery, but polymer-network interactions at the molecular level remained unresolved. The review by Firozjaii et al. [27] noted that most physical experiments and simulations are confined to reservoir and core scales, with limited microscopic analysis. Cao et al. [28] employed molecular dynamics (MD) to probe asphaltene-polymer interactions for viscosity reduction, but multivariable composite systems were not addressed. Pu et al. [29] discussed the role of HPAM-stabilized N_2_ foams from a nanostructural perspective, while quantitative links between network morphology and molecular causality were limited. Almeida et al. [30] characterized PAM and PEI hydrogels using transmission electron microscopy (TEM) and scanning electron microscopy (SEM), and reported improvements in their thermal and mechanical properties, but lacked quantitative metrics at the molecular scale. Overall, single-modality approaches (imaging alone or simulation alone) are still insufficient to explain how HPAM-surfactant-alkali formulations reshape network microstructures and their molecular drivers, and they cannot provide a transferable mechanistic map [31,32,33,34,35].

**Figure 1:**
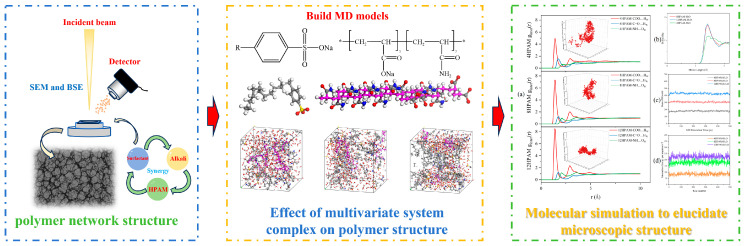
Research workflow diagram.

Focusing on polymer flooding as well as surfactant-polymer (SP) and alkali-surfactant-polymer (ASP) conditions, this study adopts HPAM, C14–LAS, and NaOH as a representative system and develops a cross-validated experimental-simulation framework. Under ambient conditions, scanning electron microscopy and backscattered electron imaging (SEM and BSE) combined with image segmentation are used to quantify network compactness and backbone integrity [36,37,38,39]; in parallel, molecular dynamics (MD) calculations of hydrogen bond statistics, radial distribution functions (RDF), and interaction energies are employed to identify the transition from hydrogen bond dominated to electrostatically dominated regimes [40,41,42,43,44]. The two evidence streams are integrated at the results stage to establish quantitative links between formulation composition, microstructural responses, and molecular interactions (Figure 1). Compared with previous studies, this work integrates scanning electron microscopy (SEM) and backscattered electron (BSE) with molecular dynamics (MD) within the same system, covering the parameter space of polymer-surfactant-alkali interactions. It reveals the bifurcation phenomenon of surfactant dosage (intercalation at low dosage versus polymer framework coverage at high dosage) and the alkali-induced ion-dominated relaxation mechanism, and it cross-validates between morphology and molecular statistics. On this basis, we provide criteria for SP and ASP formulation selection and operating-window determination in high water-cut reservoirs, offering methodological and parameterized support for optimizing chemical flooding schemes.

## 2. Experimental Program

Physical experiments (e.g., scanning electron microscopy (SEM) and backscattered electron (BSE) imaging) and molecular dynamics (MD) simulations complement each other in the study of polymer composite systems. SEM is used to visualize the network backbone structure (e.g., pore sizes and backbone thicknesses) of the HPAM composite system, whereas BSE provides localized analyses of the distributions of the chemical agents (e.g., NaOH and C14–LAS), revealing regions of chemical component interaction. MD simulation quantify the microscopic mechanisms of a complex system by analyzing intermolecular interaction energies. The trends in network morphology observed experimentally can be rationally explained by the simulated changes in the number of hydrogen bonds, as well as electrostatic and hydrophobic interactions. The combination of experimentation and computerized modeling reveals the synergistic effects of chemical agent compounding at the levels of microscopic morphology and molecular mechanisms. These findings provide strong support for the enhancement of oil displacement systems.

### 2.1. Physical Experiments

#### 2.1.1. Experimental Materials

The polymer used in this study was a polyacrylamide (HPAM) with a molecular weight of approximately 25 million (Produced by Huasheng Chemical Reagents Co., Ltd., Tianjin, China); HPAM has multiple advantages, including readily available raw materials, simple synthesis, low cost, and easy large-scale industrial production. In addition, it exhibits excellent thickening performance and is the main polymer used in water flooding applications [45]. The surfactant was C14–LAS (C14-Linear Alkylbenzene Sulfonate) (effective solid mass fraction of 40%, Supplied from Daqing Oilfield, Daqing, China); the alkali was NaOH, analytically pure (purity greater than 99.9%, Supplied from Daqing Oilfield, Daqing, China); the water used in the experiments was artificially prepared simulated formation water with mineralization of 4040.12 mg/L (Table 1). The formulations designed in this study are based on effective mass percentages.

#### 2.1.2. Instruments and Devices

Equipment included an electronic balance (Xiulab, Shanghai, China), a rotational viscometer (Brookfield, Middleboro, MA, USA), and a cold-field emission scanning electron microscope (Hitachi SU8220, Hitachi, Tokyo, Japan).

#### 2.1.3. Experimental Methods

1. The following section describes the preparation of solutions. A series of solutions was prepared using HPAM dry powder with a molecular weight of approximately 25 million. The samples were prepared by varying the polymer concentration and by adding a surfactant (C14–LAS) or an alkali (NaOH). The formulations were based on ratios commonly used in oilfield operations, and all concentrations are expressed as effective mass percentages. As shown in Table 2, the preparation of test solutions 1–14 was as follows:

2. The sample preparation process is a critical part of the overall protocol. Initially, the test samples were thoroughly homogenized. A defined volume was then dispensed with a pipette and evenly deposited onto a glass slide. The sample was prepared in a vacuum at room temperature and then coated with carbon by sputtering. Notably, this procedure accentuates the inherent polymer network, enabling, to some extent, estimation of the load-bearing limits across formulations of different concentrations. This in turn facilitates evaluation of network stability and the mechanical behavior of the polymer system under stress. We can also observe fracture in the weaker regions of the network, providing valuable insight into the polymer’s performance in practical applications. Despite the presence of certain artifacts, the method remains effective for assessing the stability of polymer networks across concentration regimes.

3. This study is observational in nature. The microstructure of the composite system in the test samples was examined by scanning electron microscopy (SEM) using backscattered electron (BSE) and secondary electron (SE) imaging modes.

### 2.2. Model Building and Simulation Methods

#### 2.2.1. Model Building

**Figure 2:**
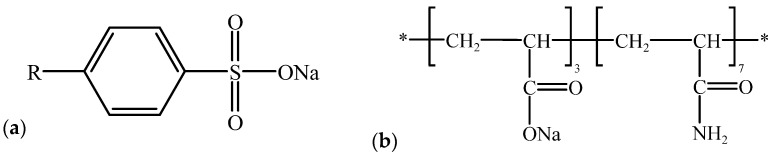
The structures of (**a**) C14–LAS (R = C14) and (**b**) HPAM monomer (* represents the polymerization connection position).

An amorphous model containing water molecules and varying numbers of polymer chains, as well as their composite systems, was constructed in Materials Studio 2023 (Amorphous Cell module) with a target density of 0.8 g/cm^3^. The molecular structure of the polymer (HPAM) was built using the Visualizer module (Build Polymers tool). Because molecular weight influences polymer-water interactions [45,46], HPAM with a degree of polymerization of 20 and a degree of hydrolysis of 30% was selected. The surfactant was modeled as C14-linear alkylbenzene sulfonate (C14–LAS), and sodium hydroxide (NaOH) was used as the alkali to provide hydroxide ions (OH^−^). Charge neutrality of the configured systems was ensured by adding sodium counter-ions (Na^+^).Its chemical formula is shown in Figure 2.

Many existing simulation experiments have demonstrated [47,48,49,50,51,52,53,54] that geometrically optimized polymer chains, C14–LAS, sodium hydroxide ions, and water molecules were randomly packed into Amorphous Cell (AC) boxes under periodic boundary conditions (PBCs). HPAM is a polymer whose oil-displacement performance depends on chain entanglement, cross-linking, and interchain interactions; selecting only a single chain would not adequately represent the three-dimensional network formed by HPAM in solution. Accordingly, the model incorporates multiple chains to better approximate the actual system and to enable the simulation of microscopic behaviors such as electrostatic repulsion, hydrogen bonding, and physical cross-linking between chains. To balance computational cost and representativeness, the base model employed four polymer chains and 400 water molecules, and the composite systems were subsequently adjusted to cover a range of concentrations.

#### 2.2.2. Simulation Methods

Water molecules were modeled using the SPC water model, and partial atomic charges for all species were assigned according to the chosen force field. For each system, ten independent initial conformations were generated, and non-bonded interactions were treated with the atom-based scheme to reduce computational cost and improve modeling efficiency. Each system was then subjected to Smart energy minimization, and the lowest-energy conformation was taken as the starting structure. The optimized amorphous polymer models were annealed for 25 cycles, heating from 300–800 K and subsequently cooling back to 300 K in 50 K increments. All molecular dynamics simulations were performed in the NPT ensemble. This annealing protocol effectively removes local conformational irregularities introduced during model building and yields more reasonable equilibrated structures for subsequent MD simulations.

To accelerate equilibration, molecular dynamics simulations were performed in the Forcite module using the COMPASS III force field in the NVT ensemble at 298 K for 500 ps. After equilibration, trajectories were sampled and used for subsequent analysis [24,55,56].

## 3. Results and Discussion

### 3.1. Effect of HPAM Concentration on Microstructure

**Figure 3:**
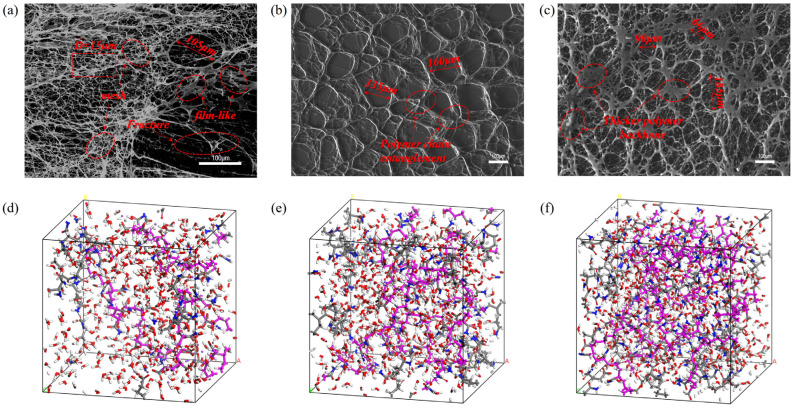
Scanning electron micrographs of HPAM at different concentrations and the corresponding amorphous polymer models are presented. The images depict the following concentrations: (**a**) 1000 mg/L, (**b**) 1500 mg/L, (**c**) 1800 mg/L, (**d**) an amorphous model constructed by four polymer chains, (**e**) an amorphous model constructed by eight polymer chains, (**f**) an amorphous model constructed by 12 polymer chains.

**Figure 4:**
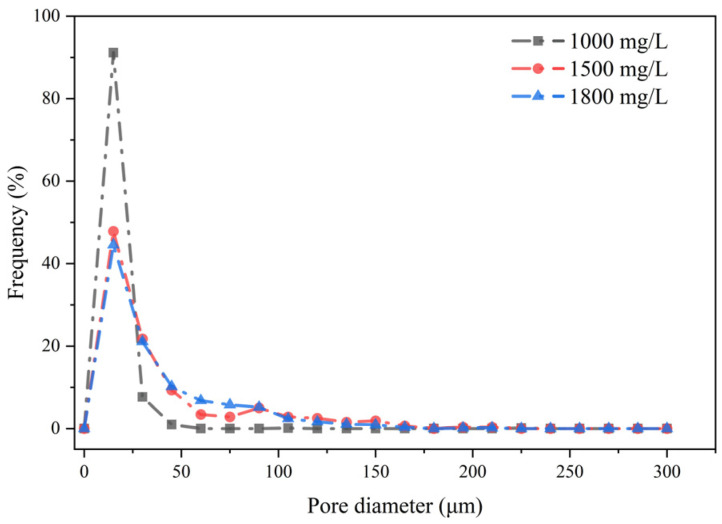
Pore size distribution images of HPAM samples with different concentrations under electron microscopy.

After carbon vacuum deposition, scanning electron microscopy images of HPAM at different concentrations were obtained (Figure 3a–c). The polymer concentrations represented in the images correspond to 1000 mg/L, 1500 mg/L, and 1800 mg/L, respectively. The pore size distribution is shown in Figure 4.

An amorphous polymer model was constructed containing 400 water molecules, with systems at varying concentrations generated by changing the number of polymer chains. Based on experimental validation, models comprising 4, 8, and 12 polymer chains were selected, and the lowest-energy conformation was taken as the initial structure. After annealing, geometry optimization, and molecular dynamics simulations, the equilibrated amorphous structures are shown in Figure 3d–f. The color scheme is: oxygen (red), hydrogen (white), carbon (gray), nitrogen (blue), and polymer chains (pink).

**Figure 5:**
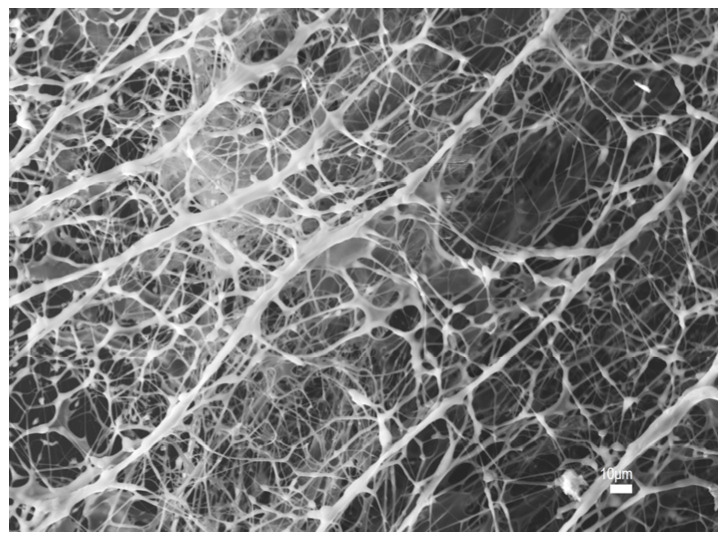
Electron microscopy image of 1000 mg/L HPAM at 330× magnification.

As shown in Figure 3a, the 1000 mg/L HPAM polymer exhibits a structure characterized by a framework or crust-like configuration, with the edges near the glass slide, while the central network is observed to float above the top of the slide. The HPAM used in this experiment is a partially hydrolyzed polyacrylamide, which is a linear polymer. In solution, HPAM adopts an extended conformation, forming a physically cross-linked spatial network structure. This is consistent with the SEM image of 1000 mg/L HPAM observed by Xiaolong Chen [57] in his study on the enhanced oil recovery potential of surfactant–polymer systems. At low concentrations, partial entanglement occurs between the linear chains, but the majority of the main chain conformations remain distinguishable, resulting in a more widely distributed fine mesh structure. Capillary forces cause the network structure to be loosely packed; the framework is fragile and prone to breakdown, as shown in Figure 5.

As shown in Figure 3b and Figure 4, at a concentration of 1500 mg/L, the network structure exhibits noticeable contraction. As illustrated in Figure 3a, the number of network pores with diameters smaller than 20 μm decreases sharply compared with the 1000 mg/L sample. This is attributed to the increased degree of polymer chain entanglement and the thickening of the network framework, which enhance the overall mechanical properties of the polymer. Consequently, the pore size distribution gradually becomes more uniform and stable.

As shown in Figure 3c and Figure 4, increasing the concentration to 1800 mg/L further strengthens the network structure. The pore-size distribution differs little from that at 1500 mg/L, indicating a stable pore distribution. However, the network density increases, and the degree of interconnection within the framework becomes more pronounced.

Consistent with previous studies [58,59,60], we employed molecular dynamics (MD) simulations to investigate the effect of HPAM concentration on the polymer microstructure. MD trajectories at different concentrations were analyzed to determine the spatial distribution probabilities between specific atoms or functional groups, the three-dimensional distribution of water molecule trajectories, and the intermolecular hydrogen-bonding and electrostatic interactions. This analysis elucidates how HPAM concentration modulates the polymer microstructure. The radial distribution function (RDF), g(r), was calculated as follows:

$$g(r)=\frac{dN}{\rho 4\pi r^{2}dr}$$

The radius of a selected range, *r*, is expressed in nanometers. The number of target atoms within a distance (*r* + d*r*) from the center atom is denoted by *N*. The average density of target atoms is represented by g/cm^3^.

**Figure 6:**
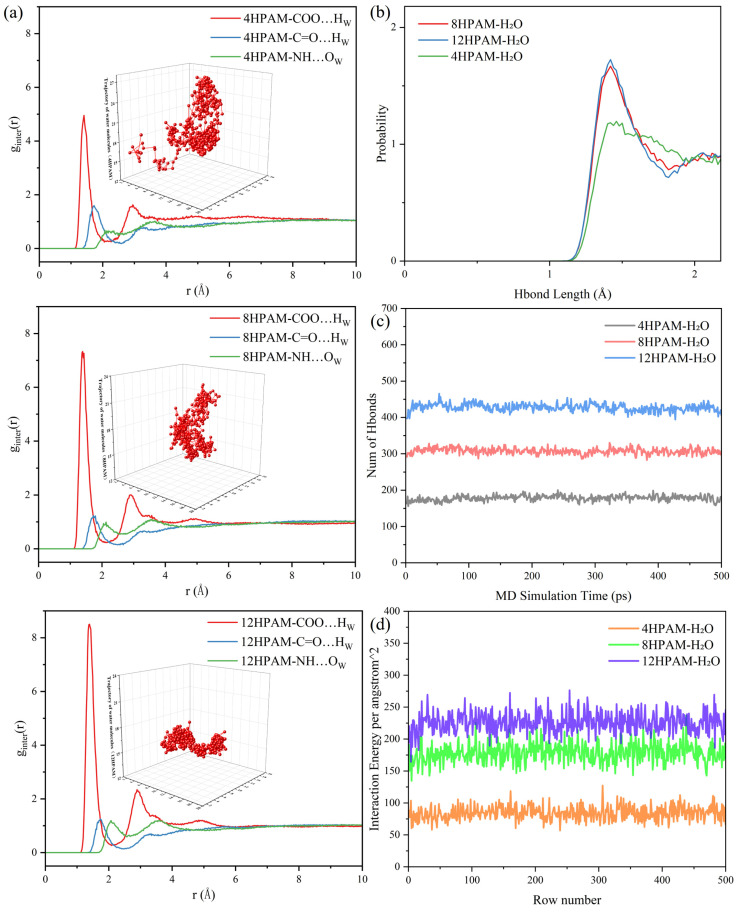
The results of the simulation and analysis of molecular dynamics of polymers with different concentrations are presented. The following aspects are examined: (**a**) a comparison of RDF of models with different polymer concentrations and a comparison of trajectories of water molecules, (**b**) a probability distribution of hydrogen bond lengths of models with different polymer concentrations, (**c**) a comparison of the number of hydrogen bonds of models with different polymer concentrations, and (**d**) a comparison of the interaction energies of the polymer molecule chains with water molecules of models with different polymer concentrations.

As shown in Figure 6a–d, the radial distribution functions (RDFs) between water molecules and HPAM chain segments increase progressively with the number of HPAM chains (4-, 8-, and 12-chain systems). The peak intensities for COO···H_w and NH···O_w likewise increase, with the NH···O_w pair exhibiting the most pronounced change. This indicates that the propensity of carboxylate oxygen atoms to form hydrogen bonds with water molecules strengthens as HPAM concentration increases. The hydrogen bond (H-bond) distributions further characterize HPAM-water interactions at different concentrations: higher HPAM concentration enhances hydrogen bonding, increases the number of hydrogen bonds, and raises the probability of a peak hydrogen bond length of approximately 1.4 Å. Concomitantly, the network becomes denser and the diffusivity of water molecules decreases, consistent with the water molecule trajectories shown in Figure 6a.

The above findings indicate that, at low concentrations (4-chain model), water molecules are primarily distributed within larger network pores and do not form strong interactions with polymer chain segments. The hydrogen bond lengths exhibit a broad and dispersed distribution. When a small external force is applied, some crosslinking points become damaged. According to the principle of energy minimization, the damaged chain segments tend to contract toward nearby crosslinked chains. Because the number of contracted chains is relatively small, the mesh radius becomes correspondingly finer. Under continuous external force, the network structure continues to deteriorate, leading to the rupture of existing networks, as shown in Figure 3a. As the fracture process progresses, the number of damaged chain segments increases, accelerating the contraction. At this stage, the larger meshes are composed of more chain segments, resulting in a relatively coarser texture. It is evident that the formation of film-like and network structures of varying sizes within the field of view arises from differences in the applied external forces and the corresponding variations in bond energy.

Such behavior arises from chain entanglement and branch-like junctions formed by physical entanglement together with ionic associations and hydrogen bonding among species in solution, rather than true covalent branching. The macroscopic reticulation thus presents a clearer, backbone-like microstructure and a more regular network skeleton. As shown in Figure 6b–d, higher polymer concentration also increases the number of hydrogen bonds, with a predominant length around 1.4 Å. This results in an increase in intermolecular forces, leading to a more compact polymer network and an increase in the thickness of the molecular framework. This plays a crucial role in stabilizing the network structure. As the network structure becomes increasingly complex and stable, the mechanical properties are enhanced, which, to some extent, leads to an increase in the polymer viscosity at the macroscopic physical level [61,62,63,64,65].

### 3.2. Effect of Anionic Surfactant (C14–LAS) Concentration on Polymer Structure

The surfactant–polymer (SP) binary system employed in this study is a commonly used oil-displacement formulation in oilfield development. The polymer is an anionic HPAM, and the surfactant C14–LAS is also anionic. In surfactant solutions, the amphiphilic molecules orient at fluid interfaces to form ordered interfacial layers, thereby exhibiting surface activity. In polymer solutions, surfactants modulate interfacial and bulk properties; conversely, polymer chain conformation and viscoelasticity influence surfactant adsorption and arrangement at interfaces, altering the overall system response.

**Figure 7:**
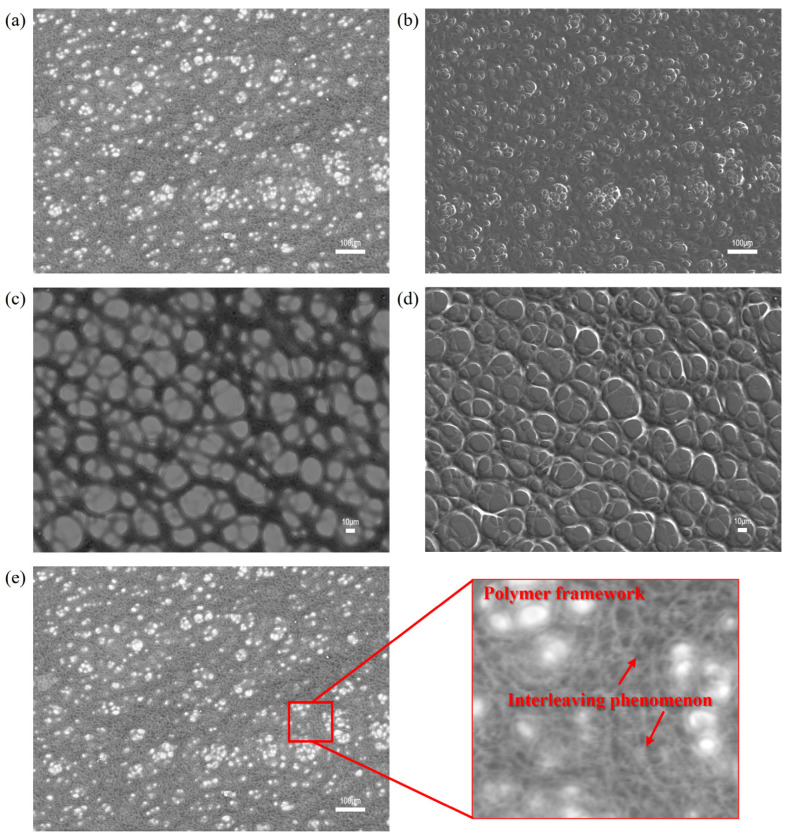
Electron microscopy and backscattered-electron images of binary systems with different concentrations of (**a**) HPAM: 1800 mg/L, C14–LAS: 0.3% backscattering image, (**b**) HPAM: 1800 mg/L, C14–LAS: 0.3% electron microscopy image, (**c**) HPAM: 1500 mg/L, C14–LAS: 0.5% backscattering image, (**d**) HPAM: 1500 mg/L, C14–LAS: 0.5% electron microscopy image; (**e**) Illustration of Interleaving Phenomenon.

The effect of polymer concentration on the microstructure was shown in Section 3.1. Building on that, we now examine the synergistic interactions between the polymer and surfactant. Mixing an anionic surfactant with an anionic polymer introduces electrostatic repulsion that markedly affects the network structure. Here, we examine the microstructure of C14–LAS/HPAM at different surfactant concentrations using scanning electron microscopy (SEM) and backscattered electron (BSE) imaging for comparison. In parallel, amorphous polymer models (Section 2) were used to support the interpretation. Figure 7 presents the SEM and BSE images of the C14–LAS/HPAM systems.

**Figure 8:**
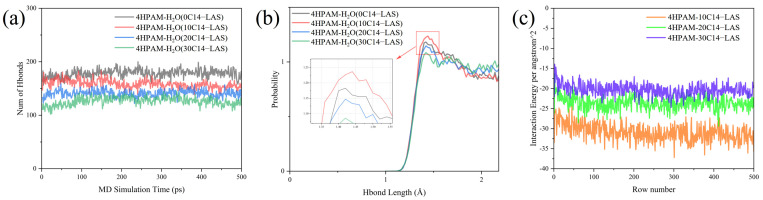
The results of the molecular dynamics simulation analysis with different concentrations of C14–LAS demonstrate the following: (**a**) a comparison of the number of hydrogen bonds for models with different C14–LAS concentrations, (**b**) a probability distribution of hydrogen bond lengths for models with different C14–LAS concentrations, and (**c**) a comparison of the interaction energies of polymer molecule chains with polymer molecule chains for models with different C14–LAS concentrations.

In accordance with the amorphous polymer model outlined in Section 3.1, the 4-chain HPAM system was selected, and C14–LAS was introduced at three loadings (10, 20, and 30 molecules). Changes in hydrogen bonding around HPAM chains and the HPAM/C14–LAS interaction energies following surfactant addition were investigated by molecular dynamics (MD) simulations, as shown in Figure 8.

As shown in Figure 7 and Figure 8, upon mixing small amounts of C14–LAS with HPAM, increasing the C14–LAS concentration reduces the polymer hydrogen-bond population relative to the surfactant-free control by 8.8% (10 LAS), 14.7% (20 LAS), and 29.4% (30 LAS) (Figure 8a). However, the short-range hydrogen-bond component at 1.3–1.6 Å attains its maximum at 10 LAS, while the position of the principal peak remains essentially unchanged (Figure 8b), indicating that under low-dosage conditions the relative fraction of strong hydrogen bonds increases whereas the total count decreases only slightly. The interaction-energy analysis shows that chain-chain attraction is strongest at 10 LAS and then progressively weakens with further dosage; relative to 10 LAS, the chain–chain interaction energy at 20 and 30 LAS increases by 25.5% and 40.4% (i.e., becomes less negative, reflecting weaker attraction) (Figure 8c). These results suggest that small amounts of C14–LAS can intercalate into the polymer structure and raise the local charge density, thereby enhancing the effective hydrogen-bonding capacity of HPAM even as the overall hydrogen-bond number declines slightly; this is consistent with the assessment that electrostatic repulsion is negligible at low dosage, permitting surfactant incorporation.

Morphological observations further corroborate this mechanism: under 10 LAS, the framework forms a continuous network, with bright BSE signal distributed along the skeleton (Figure 7b versus Figure 7a), indicating surfactant enrichment in the vicinity of the framework and interpenetration with the polymer. When the surfactant proportion is increased further (Figure 8c), chain-chain attraction weakens further and electrostatic repulsion strengthens, and a fraction of water molecules depart from the HPAM hydration shell to form hydrogen bonds with C14–LAS; interpenetration is consequently attenuated and partial coverage of the framework emerges (Figure 7a,e). Taken together, these results indicate that the relative surfactant concentration governs its preferred adsorption sites and configurations within the polymer system, thereby significantly affecting the oil-displacement and interfacial-tension-reduction performance of the HPAM/C14–LAS binary system in engineering applications [21,66,67,68,69].

### 3.3. Effect of Alkali/Surfactant on Polymer Microstructure

#### 3.3.1. Effect of Crystallization

**Figure 9:**
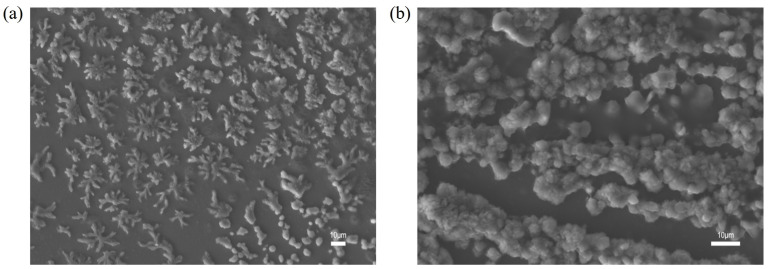
Scanning electron microscopy images of the NaOH/C14–LAS/HPAM composite system at (**a**) 500× and (**b**) 1000× magnification.

The alkali–surfactant–polymer (ASP) ternary composite system represents a significant advance in chemical EOR, leveraging synergistic interactions among its constituents to combine the advantages of alkali, surfactant, and polymer, thereby achieving notable improvements in oil recovery [70]. In this study, the alkali was sodium hydroxide (NaOH), the surfactant was C14-linear alkylbenzene sulfonate (C14–LAS), and the polymer was an anionic HPAM. Representative microstructures of the NaOH/C14–LAS/HPAM system are shown in the scanning electron microscopy (SEM) images in Figure 9.

When NaOH is dissolved in the system, the added alkali partially hydrolyzes the polymer. During sample preparation for SEM, vacuum drying leads to water removal and supersaturation, causing crystallization of NaOH and other salts. This crystallization and nucleation can disrupt the spatial network, rupture the original structure, and produce isolated crystalline nuclei or stripe-like (lamellar) features. However, under conventional oilfield operating conditions, the addition of alkali generally does not induce bulk crystallization; the above features are attributable to sample-preparation artifacts. In the following analysis, we examine these experimental observations and, using molecular dynamics (MD) simulations, investigate the effect of alkali addition on the composite system. In the simulations, alkali was introduced after equilibrating the surfactant-polymer (SP) system to form the alkali-surfactant-polymer (ASP) system.

#### 3.3.2. Molecular Dynamics Analysis of Ternary Systems

**Figure 10:**
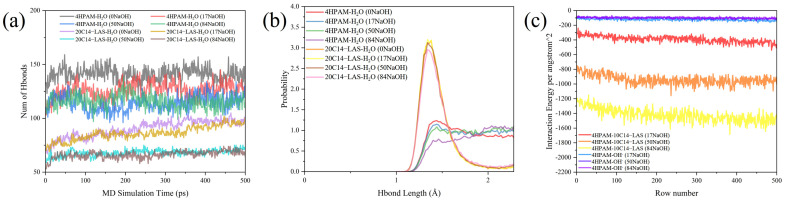
The results of the molecular dynamics simulation analysis of composite systems with different concentrations are presented. The analysis includes the following aspects: (**a**) a comparison of the number of hydrogen bonds, (**b**) a probability distribution of hydrogen bond lengths, and (**c**) a comparison of interaction energies.

Building on the preceding sections, the composite system comprising four HPAM chains and 20 C14–LAS molecules was selected. Microstructural changes were probed by molecular dynamics (MD) simulations by introducing NaOH at three loadings (17, 50, and 84 NaOH ion pairs per simulation cell). We analyzed (i) the numbers and length distributions of hydrogen bonds between HPAM and water and between C14–LAS and water, and (ii) the pairwise interaction energies between HPAM and C14–LAS and between HPAM and OH^−^ at each alkali loading. The results are shown in Figure 10.

With increasing alkali concentration, the total number of hydrogen bonds between the polymer and water decreases overall; the decline is most pronounced at low alkali dosages and then gradually levels off (Figure 10a). Correspondingly, in the hydrogen-bond-length distribution, the probability at approximately 1.4 Å decreases continuously, the distribution shifts toward longer bond lengths, and the fraction at lengths greater than 1.4 Å increases (Figure 10b), indicating a weakening of polymer-water attractive interactions. This occurs because OH^−^ preferentially associates with water to form hydrogen bonds, competitively occupying sites that would otherwise be available to the polymer and the surfactant, thereby reducing their hydrogen bonds with water in parallel. Nevertheless, the sulfonate polar headgroup (-SO_3_^−^) of C14–LAS is strongly hydrophilic and can still form stable hydrogen bonds with hydrogen-bond donors in water (-H); accordingly, the LAS-water hydrogen-bond lengths remain concentrated around approximately 1.4 Å.

In conjunction with Figure 10c, the intermolecular attraction between HPAM (partially hydrolyzed polyacrylamide) and C14–LAS strengthens progressively as the alkali concentration increases; meanwhile, the HPAM-OH^−^ interaction energy is markedly weaker than that of HPAM-LAS. As the involvement of OH^−^ rises, the net HPAM-OH^−^ attraction diminishes further while electrostatic repulsion becomes more pronounced, which constrains additional association between HPAM and OH^−^. Considering the hydrogen-bond statistics together with the changes in intermolecular interaction energies, we infer that the introduction of alkali gradually loosens the network, disperses the chain segments, and enlarges certain pores, thereby weakening physical entanglements and chemical cross-linking among polymer segments. Consequently, the microstructure becomes more distinctly ion-dominated rather than a dense network governed primarily by direct polymer–polymer interactions.

## 4. Conclusions

### 4.1. Results

1. Increasing the HPAM concentration enhances the network density and stability. Both SEM and molecular dynamics (MD) simulations indicate that higher HPAM concentrations increase hydrogen bonding interactions with water molecules, resulting in the formation of a more stable and compact network. This microscopic structural change strengthens the tightness of the polymer solution network and, to some extent, explains the increase in polymer viscosity, which is attributable to enhanced intermolecular interactions and reduced intermolecular distances, thereby increasing the flow resistance of the solution.

2. The effect of surfactant concentration on the polymer is mainly governed by electrostatic repulsion. When the surfactant concentration is low, the electrostatic repulsion in the system is weak, and a small amount of C14–LAS can penetrate the polymer structure. The polymer and surfactant molecules intercalate with each other, which also explains why a small amount of surfactant has limited effect in practical applications. However, as the surfactant concentration increases and reaches a level sufficient to generate significant electrostatic repulsion, the surfactant molecules aggregate and cover the polymer surface due to electrostatic repulsion.

3. The addition of alkali makes the network structure looser, resulting in a greater number of dispersed chain segments and enlarging some network pores. This weakens the physical entanglement or chemical crosslinking between polymer chains and leads to the development of more pronounced ion-dominated microscopic structural characteristics in the system.

### 4.2. Limitations of the Study

1. This study did not use the freeze-vacuum sectioning method, so the polymer network structure observed may differ from the true in situ structure. The aim was to examine the mechanical rupture characteristics of the polymer network for practical oilfield applications. However, this method has limitations in accurately reflecting the true in situ structure.

2. Experimental conditions (e.g., temperature and salinity) were kept constant. While this approach isolates the effects of polymer concentration, surfactants, and alkalis, it may not fully replicate the complex and variable conditions in real oilfields.

3. Molecular dynamics simulations employed a simplified polymer network model. While it captures basic interactions between the polymer, surfactants, and alkali, it does not account for complexities like varying HPAM molecular weights, different ions, or dynamic changes in solution conditions, meaning the results may not fully reflect the behavior of the polymer system under real-world conditions. A comparison table with existing studies for analysis, as shown in Table 3.

### 4.3. Future Research Directions

1. Future research should explore the behavior of polymer flooding systems under more complex and variable conditions, such as high temperatures, fluctuating salinity, and the presence of different oils. These factors significantly affect the performance of polymer networks, and incorporating them will provide a more comprehensive understanding of polymer systems in real oilfield environments.

2. Advanced imaging techniques, such as cryo-SEM, should be employed to better preserve the in situ 3D network structure of polymers in their hydrated state. This would enable more accurate characterization of polymer networks under conditions closer to those found in actual oilfields.

3. Expanding molecular dynamics simulations to include more complex models, such as those with varying HPAM molecular weights, different ionic species, and dynamic reservoir conditions, will offer deeper insights into the intermolecular interactions within polymer systems and improve the predictive capability of simulations in real-world applications.

4. Future studies should focus on developing environmentally friendly and sustainable polymer systems for enhanced oil recovery (EOR). This includes investigating the use of biodegradable polymers and eco-friendly surfactants to reduce the environmental impact of polymer flooding technologies.

## Figures and Tables

**Table 1:** Simulated ionic composition of formation water.

Mineral Ions	Na^+^	Ca^2+^	Mg^2+^	HCO_3_^−^	Cl^−^	SO_4_^2−^	CO_3_^2−^
Concentration (mg/L)	1277.22	31.92	7.35	1705.62	798.29	9.65	210.07

**Table 2:** Formulations of test solutions.

Number	Polymer (25 Million Molecular Weight HPAM Dry Powder)	Surfactant (C14–LAS)	Alkali (NaOH)
1	1000 mg/L	--	--
2	1500 mg/L	--	--
3	1800 mg/L	--	--
4	1800 mg/L	0.6 wt% a.i.	--
5	1800 mg/L	0.3 wt% a.i.	--
6	1500 mg/L	0.5 wt% a.i.	--
7	1800 mg/L	0.3 wt% a.i.	0.2 wt% a.i.
8	1800 mg/L	--	0.3 wt% a.i.
9	1800 mg/L	0.2 wt% a.i.	0.3 wt% a.i.
10	1800 mg/L	0.4 wt% a.i.	0.3 wt% a.i.
11	1800 mg/L	0.6 wt% a.i.	0.3 wt% a.i.
12	1800 mg/L	0.8 wt% a.i.	0.3 wt% a.i.
13	1800 mg/L	1.0 wt% a.i.	0.3 wt% a.i.
14	1800 mg/L	1.2 wt% a.i.	0.3 wt% a.i.

**Table 3:** Comparison of representative prior studies and the present work.

Study (Ref.)	System & Scope	Methods/Scale	Key Result	Main Limitation vs. Present Aims
Li et al. [25]	Polymer systems; evaluation of polymer with alkali and surfactant (ASP)	Macro-scale tests and modeling	Enhanced viscoelasticity and interfacial activity; ASP performance assessed	Composite-network microstructure not quantified; no experiment-simulation linkage
Li et al. [26]	ASP flooding; visual micromodels	Pore-scale visualization	ASP synergy; recovery improvement reported	Molecular-level interactions within the polymer network unresolved; no SEM and BSE-MD integration
Firozjaii et al. [27]	Review of polymer flooding	Literature synthesis (reservoir and core scales emphasized)	Field-relevant progress summarized	Microscopic mechanisms underanalyzed; no transferable microstructure-mechanism map
Cao et al. [28]	Asphaltene-polymer interactions	MD (molecular dynamics)	Viscosity-reduction mechanisms discussed	Not a multivariable composite; lacks linkage to electron-microscopy network evidence
Pu et al. [29]	HPAM-stabilized N_2_ foams	Nanostructural observations	Improved sweep with HPAM-stabilized foams	Limited quantitative metrics for network morphology; molecular causality not established
Almeida et al. [30]	PAM and PEI hydrogels	TEM and SEM morphology; property tests	Thermal and mechanical enhancements	Different material system; lacks molecular-scale quantitative analysis
This work	HPAM/C14–LAS/NaOH; SP, ASP and polymer flooding conditions	Integrated SEM and BSE (ambient) + MD (H-bonds, RDF, interaction energies)	Within a single system, SEM and BSE are coupled with MD and cross-validated by morphology and molecular statistics.	Addresses above gaps via cross-validation of morphology and molecular statistics; provides actionable criteria for SP and ASP formulation and operating-window selection

## Data Availability

The original contributions presented in this study are included in the article. Further inquiries can be directed to the corresponding author.

## Nomenclature

ASP	Alkali-Surfactant-Polymer	NPT	Isothermal-Isobaric Ensemble
Å	Ångström	NVT	Canonical Ensemble
AC	Amorphous Cell	PAM	Polyacrylamide
BSE	Backscattered Electron	PBC(s)	Periodic Boundary Conditions
C14–LAS	C14-Linear Alkylbenzene Sulfonate	PEI	Polyethyleneimine
COMPASS III	Condensed-phase Optimized Molecular Potentials for Atomistic Simulation Studies (version III)	RDF	Radial Distribution Function
EOR	Enhanced oil recovery	SE	Secondary Electron (imaging)
HPAM	Partially hydrolyzed polyacrylamide	SEM	Scanning Electron Microscopy
H-bond/HBond	Hydrogen bond	SP	Surfactant-Polymer
MD	Molecular Dynamics	SPC	Simple Point Charge (water model)
NaOH	Sodium Hydroxide	TEM	Transmission Electron Microscopy
NMR	Nuclear Magnetic Resonance	wt% a.i.	weight percent active ingredient
